# Clinical application of cone beam CT in intensity‐modulated radiotherapy for the moderate to severe thyroid‐associated ophthalmopathy

**DOI:** 10.1002/acm2.70416

**Published:** 2025-12-12

**Authors:** Dan Kang, Hao Hu, Shuyun Ma, Shizhen Bin

**Affiliations:** ^1^ Department of Oncology The Third Xiangya Hospital, Central South University Changsha Hunan China; ^2^ Radiotherapy Center The Third Xiangya Hospital, Central South University Changsha Hunan China

**Keywords:** cone beam computed tomography, intensity‐modulated radiotherapy, setup errors, thyroid‐associated ophthalmopathy

## Abstract

**Background:**

Cone beam CT (CBCT) has been reported for intensity‐modulated radiation therapy (IMRT) of various cancers due to its real‐time imaging capabilities, but studies on its application in orbital diseases, particularly moderate to severe thyroid‐associated ophthalmopathy (TAO), remain limited.

**Purpose:**

To investigate the clinical applications of CBCT in IMRT for TAO, with a focus on setup accuracy, dosimetric impact, and short‐term changes in eye prominence.

**Methods:**

A total of 115 CBCT scans from 17 TAO patients were analyzed. Pretreatment CBCT scans were registered to the planning CT using bony anatomy. The resulting setup errors were quantified in three translational directions. Residual shifts after online correction were tracked to assess interfractional variation and trends throughout the treatment course. Dose‐volume histogram from plans recalculated with simulated setup errors (mean and maximum values) were compared to the original plan. Eye prominence was measured on serial CBCT scans, and its correlation with cumulative dose was analyzed.

**Results:**

Mean setup errors were (–0.16 ± 1.09) mm, (–0.19 ± 1.18) mm, and (–0.55 ± 1.05) mm in X, Y, and Z directions. Errors significantly decreased planning target volume (PTV) conformity (*p* < 0.05) and increased *D*_mean_, *D*_max_, *D*_2%,_ and *D*_50%_ (*p* < 0.05). For organs at risk (OARs), maximum and mean doses increased, with left lens *D*_max_ increasing by 21.3%, 32.7%, 33.8%, and 13.2% for mean setup errors and X, Y, and Z maximum errors. Right lens showed similar increases. A correlation between CBCT‐measured eye prominence and total radiotherapy dose was observed, with the binomial fit: y = 22.43 **+** 0.12x − (9.94 × 10^−3^) x^2^ (*R^2^* = 0.96). Mean eye prominence decreased significantly from (21.74 ± 1.74) mm pre‐treatment to (20.94 ± 1.73) mm after 18 Gy of IMRT (*p* < 0.05).

**Conclusion:**

CBCT image guidance for IMRT in moderate to severe TAO improves target conformity and coverage while reducing OAR doses, particularly for lenses. CBCT also shows promise for short‐term efficacy assessment of eye prominence in radiotherapy patients which may alleviate patient anxiety with preliminary observation thereby improving patient compliance with radiotherapy.

## 1 INTRODUCTION

Thyroid‐associated ophthalmopathy (TAO) is an autoimmune disease affecting intraorbital and periorbital tissues^1^ and is the most common orbital disease in adults.^2^ Radiotherapy has emerged as an effective treatment for moderate to severe TAO, owing to its non‐specific anti‐inflammatory effects and the high radiosensitivity of lymphocytes.^3^ However, conventional radiotherapy techniques are limited by field angles, often resulting in insufficient dose delivery to the anterior globe or retro‐orbital fat when attempting to reduce lens exposure.

The advent of IMRT achieved highly conformal dose distributions.^4^ However, the full potential of IMRT was only realized with equally high‐precision patient positioning, a limitation of conventional IMRT that relied solely on skin marks for setup. Image‐guided radiation therapy (IGRT) using CBCT was developed to address this gap. Compared to these non‐IGRT protocols, daily online CBCT‐IGRT ensured accurate patient positioning immediately before each treatment, thereby improving the accuracy of every dose delivery.^5^ The clinical benefits of this technological advancement are evident in the literature. For instance, Nien et al.^6^ demonstrated the superior efficacy of IGRT, showing that it significantly enhanced overall survival and local progression‐free survival in patients with locally advanced oral cancer compared to historical controls without routine daily IGRT. Furthermore, Den et al.^7^ provided direct evidence of the precision advantage of IGRT in a prospective trial, showing that CBCT guidance allowed for a reduction of the PTV margin by ≈ 50% compared to the standard margins used without daily IGRT. This margin reduction directly facilitated dose escalation to the target and/or a reduction in toxicity to surrounding normal tissues.

While CBCT has been reported in the treatment of various cancers such as lung cancer,^8^ liver cancer,^9^ nasopharyngeal carcinoma,^10^ and so forth^11^ studies on its application in orbital diseases, particularly moderate to severe TAO, remain limited. Exophthalmos, a key symptom of moderate to severe TAO, serves as a crucial grading criterion in the NOSPECS classification system.^12^ Radiotherapy has been shown to effectively eliminate lymphocytes in orbital tissues and reduce cytokine production, thereby alleviating orbital edema and tissue fibrosis.^4^, ^13^ Clinical data indicated an immediate improvement rate of 94.5% in exophthalmos following radiotherapy, with a sustained improvement rate of 78% at 3 years post‐treatment.^14^

In typical IMRT protocols for TAO, a total dose of 20 Gy is delivered over 10 fractions. Patients may not perceive immediate improvement, particularly in the early stages of treatment, and some may experience acute local radiation reactions. These factors can impose significant psychological burden on patients and potentially lead to doubts about treatment efficacy. To address this, we propose utilizing CBCT imaging data obtained during each radiotherapy session to evaluate short‐term efficacy by measuring eye prominence values, aiming to alleviate patient anxiety with empirical clinical data. Given this clinical context, this study aims to explore the application of CBCT image‐guided IMRT for moderate to severe TAO, with a specific focus on evaluating radiotherapy positioning accuracy and short‐term changes in eye prominence.

## 2 METHODS

### 2.1 Patient selection

Seventeen cases of moderate to severe TAO patients treated with CBCT‐guided IMRT at the Radiotherapy Center were retrospectively analyzed from November 2020 to July 2025. The cohort comprised seven males and 10 females, aged 32–73 years (median: 54 years), all with bilateral involvement and normal cardiovascular and pulmonary function.

### 2.2 Target volume contouring and IMRT plan design

Patients were immobilized in the supine position using thermoplastic head masks. CT scans (1 mm slice thickness) were acquired using a Siemens large‐aperture CT scanner and transferred to the Varian Eclipse 11.0 treatment planning system. The clinical target volume (CTV) and planning target volume (PTV) were contoured according to the ICRU Reports 62^15^ and 83.^16^ Organs at risk (OARs), including the lenses, optic nerves, and optic chiasm, were contoured on the CT images. The prescribed dose was 20 Gy in 10 fractions, with > 90% of PTV receiving 100% of the prescribed dose. IMRT plans with 10–12 fields (240^°^–120^°^) were designed, and treatment was delivered using 6 MV X‐rays at 400 MU/min on a Varian TrueBeam linear accelerator.

### 2.3 CBCT image‐guided and radiotherapy delivery

Pre‐treatment kV CBCT scans were acquired using the on‐board imaging system of the TrueBeam medical linear accelerator, obtaining 3D CBCT image data of the patient's head. The specific CBCT scanning protocol was as follows: a standard head scanning mode was employed, with a tube voltage of 100 kV, tube current of 15 mA, pulse time of 20 ms, gantry rotation speed of 6°/s exposure of 150 mAs, a full fan type, 512 × 512 pixel, and 2 mm slice thickness. Automatic bone density‐based registration was used to align the planning CT with the 3D CBCT online. Clinical radiation oncologists manually adjusted the registration in axial, sagittal, and coronal planes around the target area layer‐by‐layer to achieve optimal matching of the target volumes and OARs. Finally, upon confirmation and signing by the radiation therapist, medical physicist, and radiation oncologist, the resulting couch shifts (in the X, Y, and Z directions) were recorded and applied for patient positioning correction prior to treatment delivery. CBCT guidance was performed for all TAO patients during their first and last fractions, and at least every third fraction thereafter. This fractionated image‐guided protocol, based on the actual conditions of our center, was designed to balance the need for high targeting accuracy throughout the treatment course with considerations of clinical workflow efficiency and cumulative imaging‐related radiation exposure. On treatment days without CBCT guidance, patient positioning was verified using a conventional protocol relying solely on external skin markers and laser alignment. A total of 115 CBCT‐guided treatments were conducted.

### 2.4 Dose simulation based on couch shift parameters and plan evaluation

The average and maximum setup errors in X, Y, and Z directions were input into the treatment planning system for dose simulation. Four simulated plans were generated based on average setup errors and maximum setup errors in each direction. These shifted plans were compared against the original plans to assess the impact of setup errors on dosimetric parameters for PTV and OARs. For PTV, maximum dose (*D*_max_), minimum dose (*D*_min_), mean dose (*D*_mean_), dose to 2%, 50%, 95%, 98% of volume (*D*_2%_, *D*_50%_, *D*_95%_, *D*_98%_), and Conformity Index (CI = V_100%_/V_PTV_) were evaluated. For OARs, *D*_mean_ and *D*_max_ of bilateral lenses, and D_max_ of bilateral optic nerves and optic chiasm were assessed.

### 2.5 Short‐term efficacy assessment of eye prominence

CBCT 3D images were used to measure the eye prominence in both eyes of the patients. All measurements were performed according to the following protocol: CBCT images were first adjusted to an optimal window width and level for orbital visualization (window width: 280 HU; level: 90 HU). The axial slice displaying the largest cross‐sectional area of the eyeball and optic nerve was then selected. On this slice, the perpendicular distance from the corneal apex to the interzygomatic line, defined as the line connecting the most anterior points of the bilateral zygomatic arches, was measured. To ensure measurement accuracy and consistency, all assessments were independently performed by a radiation oncologist and subsequently verified by a medical physicist. Any discrepancies exceeding 1 mm were re‐evaluated collaboratively until consensus was achieved. The bionomial fitting was performed to analyze the potential correlation between eye prominence and the radiotherapy dose, and to evaluate the application of CBCT in assessing short‐term therapeutic effects on eye prominence.

### 2.6 Statistical analysis

Paired *t*‐tests were performed using SPSS 18.0 software to compare radiotherapy plans and ocular prominence before and after treatment. Measurements were expressed as mean ± standard deviation (x̄ ± s), with *p* < 0.05 considered as statistically significant.

## 3 RESULTS

### 3.1 Analysis of setup errors based on CBCT

**FIGURE 1:**
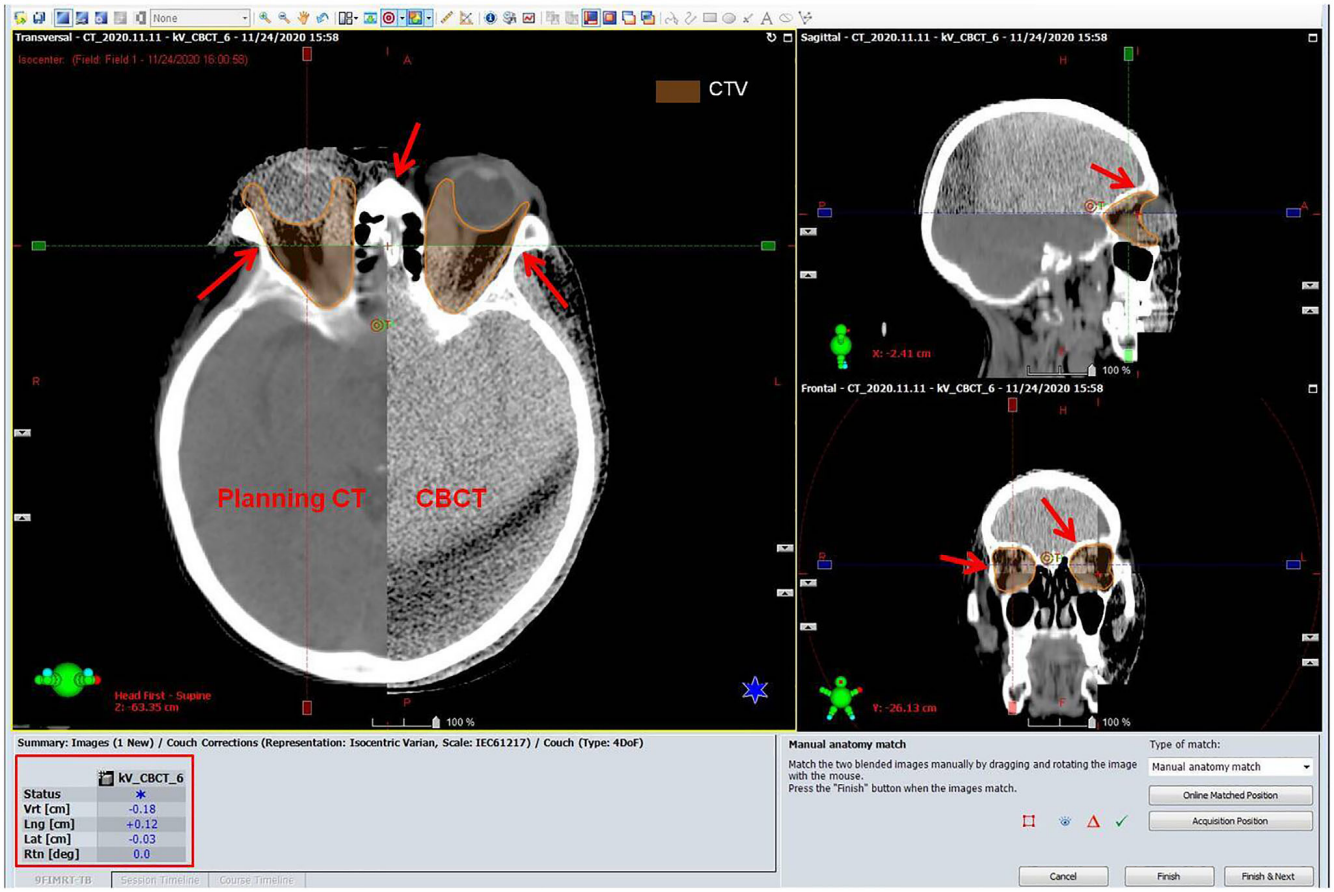
Setup errors in cone beam CT (CBCT) bony alignment correction in a patient with moderate to severe thyroid‐associated ophthalmopathy (TAO).

Figure 1 illustrates the process of correcting setup errors using bone registration after CBCT scanning in a typical patient with moderate to severe TAO. The CBCT scan images were registered with the patient's planning CT images using bone registration. Red arrows indicate the main bony landmarks around the orbit in axial, sagittal, and coronal planes. The registration results (shown in the red rectangular box) display the setup error values in the X, Y, and Z directions.

**FIGURE 2:**
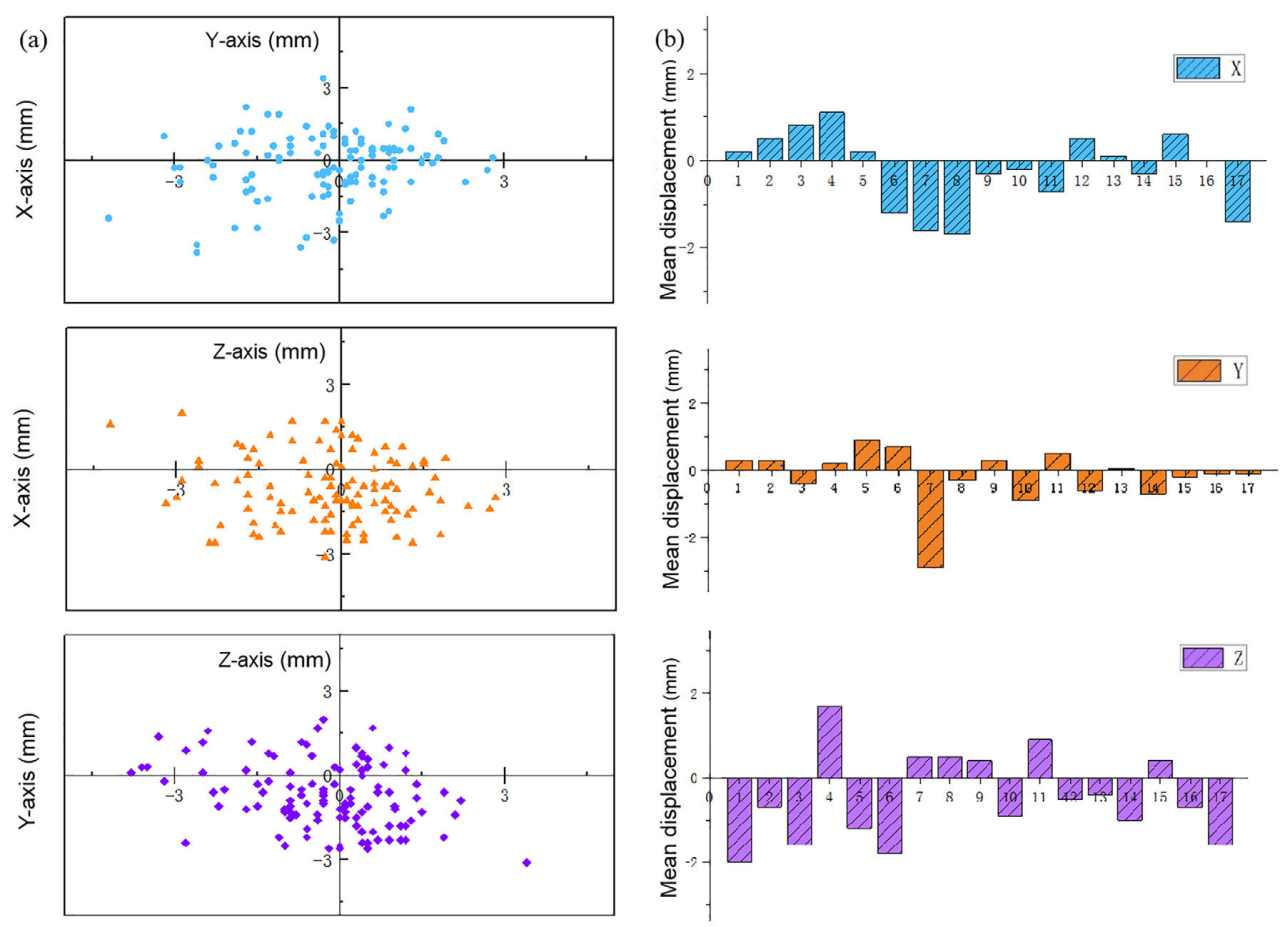
(a) Coordinate distribution of the 115 setup errors for all patients. (b) Mean setup errors in the X, Y, and Z directions.

Figure 2a presents the results of 115 setup error analyses from 17 patients with moderate to severe TAO. The frequency distribution in the left‐right X‐axis direction was approximately uniform. In the head‐foot Y direction, there was a slightly higher frequency towards the head. In the anterior‐posterior Z‐axis direction, a higher frequency towards the posterior was observed. The X‐Y plot showed that overall setup errors were centrally distributed in the first, second, and fourth quadrants, with a roughly uniform distribution in the X direction and a slightly higher frequency in the positive Y direction. The X‐Z plot revealed that overall setup errors were mainly concentrated in the first, third, and fourth quadrants, particularly in the fourth quadrant. The X direction distribution was similar to that in the X‐Y plot, while the Z direction showed a notably higher frequency in the negative direction. The Y‐Z plot demonstrated that overall setup errors were primarily distributed in the third and fourth quadrants, with the Z direction distribution similar to that in the X‐Z plot, and the Y direction showing a roughly uniform distribution.

Figure 2b displays the average displacements, which showed a similar distribution. Overall, the absolute values of the mean displacements in the negative X, Y, and Z directions were greater than those in the positive directions. The number of cases was evenly distributed in the positive and negative X directions, while the distribution was uneven in the Y and Z directions. The mean displacement of all patients in the dorsal direction was greater than that in the ventral direction.

The mean setup errors for IMRT of moderate to severe TAO were (–0.16 ± 1.09 mm), (–0.19 ± 1.18) mm, and (–0.55 ± 1.05) mm in the X, Y, and Z directions, respectively. Table 1 shows that the setup error distribution ranges for the X, Y, and Z directions were –4.2 to 2.8 mm, –3.8 to 3.4 mm, and –3.1 to 1.7 mm, respectively. The systematic errors in all directions were below 1 mm, while the random errors were all between 1 and 3 mm. The setup error distribution in the Z direction was asymmetrical, and the error deviation in the Y direction was large.

**TABLE 1:** Setup errors for all patients.

			Error extremum	
Direction	Systematic error	Random error	Minimum	Maximum
X	−0.16	1.09	−4.2	2.8
Y	−0.19	1.18	−3.8	3.4
Z	−0.55	1.05	−3.1	1.7

### 3.2 Dosimetric effects of setup errors on IMRT plans

**FIGURE 3:**
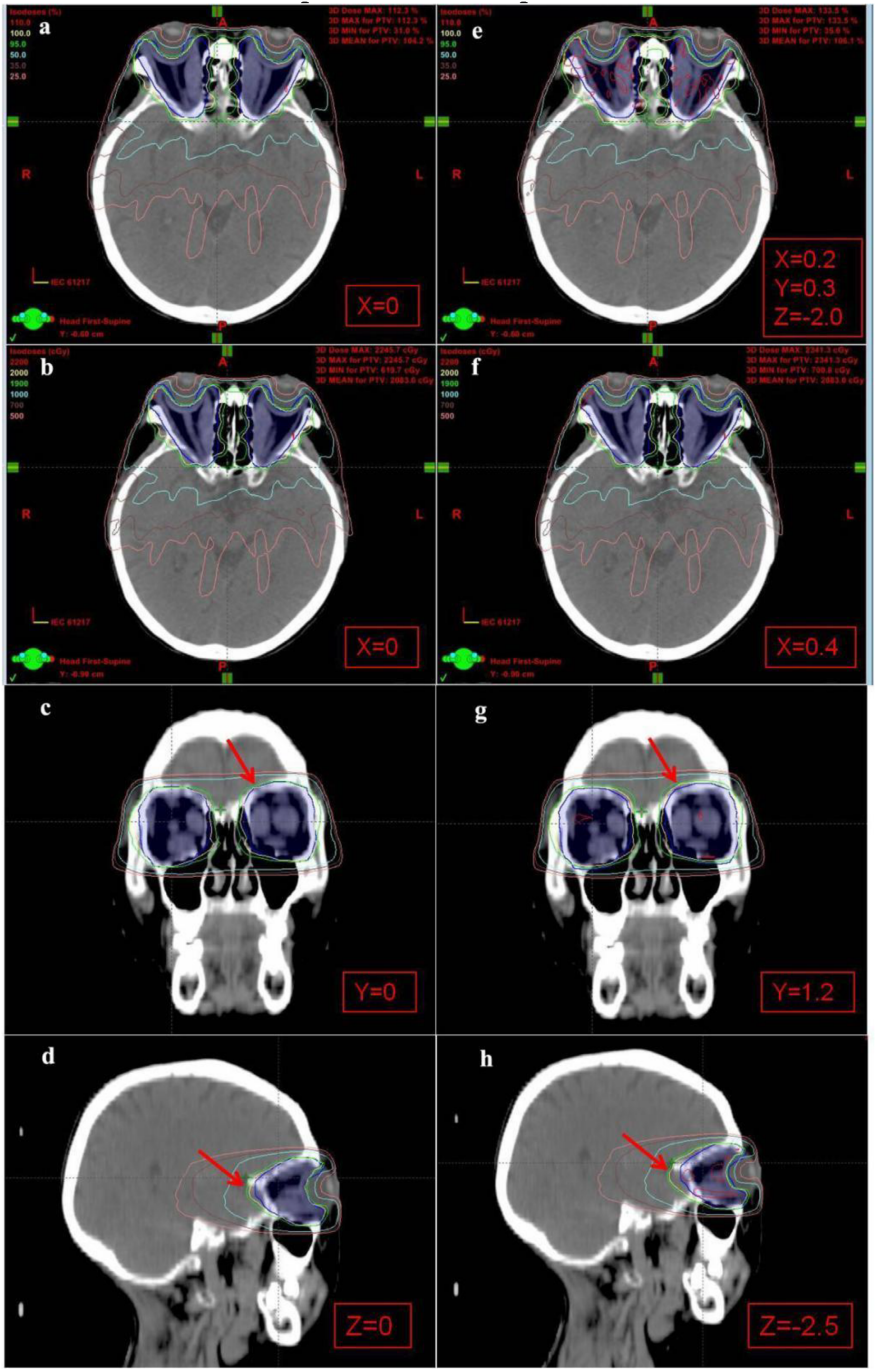
The dosimetric impact of setup errors in the X, Y, and Z directions on the plan of a thyroid‐associated ophthalmopathy (TAO) patient: (a), (b), (c), and (d) was the original plan dose. (e), (f), (g), and (h) was the shifted plan dose.

**FIGURE 4:**
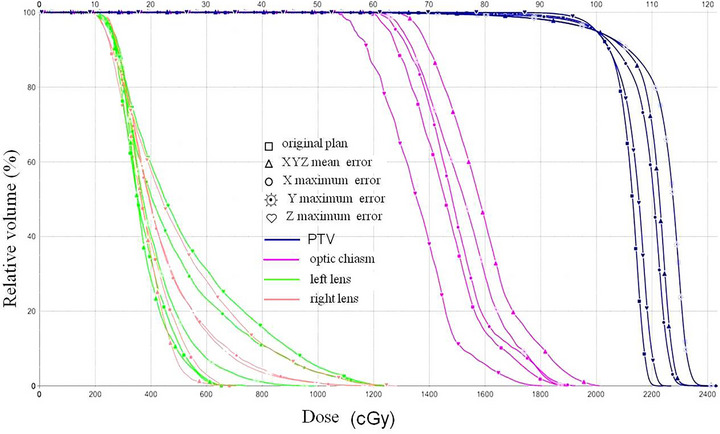
Dose volume histogram of a thyroid‐associated ophthalmopathy (TAO) patient plan affected by mean and maximum setup errors.

Figure 3 provides a visual representation of the dose distribution affected by setup errors in a typical moderate to severe TAO patient's plan. Figure 3e–h shows the plan dose distribution after applying the average errors simultaneously in X, Y, and Z directions (X = 0.2, Y = 0.3, Z = –2.0). A notable increase in the hot spots of the target area was observed. The hotspot also increased when maximum errors were applied in all single directions (X = 0.4 mm, Y = 1.2 mm, Z = –2.5 mm). With larger setup errors, such as Z = –2.5 mm, the isodose lines shifted posteriorly (indicated by red arrows), potentially leading to partial omission of the anterior retro‐ocular target area. The dose volume histogram (Figure 4) for this patient further illustrates that, under the same target dose normalization conditions, plans with average and maximum setup errors showed a significant increase in high‐dose volume to the target area compared to the original plan.

Table 2 presents the statistics of the PTV dose affected by setup errors for all patients. Under the same target dose normalization conditions, all mean and maximum couch shifts significantly affected *D*_max_, *D*_mean_, *D*_2%_ and *D*_50%_ of PTV. All of them increased, with *D*_max_ increasing by 5.2%, 5.0%, 4.4%, and 5.4%, *D*_mean_ by 2.4%, 1.9%, 2.7%, and 2.2%, *D*_2%_ by 2.8%, 2.7%, 3.7%, and 2.7%, *D*_50%_ by 2.8%, 2.4%, 3.5%, and 2.9%, compared to the original plan. The impact on PTV *D*_min_, *D*_95%_, and *D*_98%_ was not significant.

**TABLE 2:** Statistical analysis of planning target volume (PTV) dose parameters affected by setup errors (cGy) ($\bar{x}\pm s$, cGy).

Evaluation parameter	Original plan	XYZ_mean error	X_max error	Y_max error	Z_max error	*p*‐value (XYZ_mean)	*p*‐value (X_max)	*p*‐Value (Y_max)	*q*‐Value (Z_max)
PTV_*D*_min_	869.20 ± 382.82	831.48 ± 345.72	761.30 ± 332.90	831.92 ± 319.53	802.57 ± 364.34	0.423	0.000	0.410	0.135
PTV_*D*_max_	2246.94 ± 39.47	2363.78 ± 137.01	2360.30 ± 87.93	2345.12 ± 102.23	2367.25 ± 143.10	0.003	0.002	0.001	0.003
PTV_*D*_mean_	2090.85 ± 21.81	2140.89 ± 81.36	2130.23 ± 52.11	2147.80 ± 77.03	2136.71 ± 61.45	0.023	0.001	0.005	0.007
PTV_*D*_2%_	2191.88 ± 26.24	2253.07 ± 101.19	2250.01 ± 61.16	2274.05 ± 87.59	2251.43 ± 67.26	0.025	0.001	0.001	0.003
PTV_*D*_50%_	2115.39 ± 18.47	2174.85 ± 91.01	2165.55 ± 59.45	2188.95 ± 82.47	2177.25 ± 75.75	0.012	0.001	0.001	0.005
PTV_*D*_95%_	1929.17 ± 63.99	1922.75 ± 84.60	1933.68 ± 56.98	1917.97 ± 71.36	1881.59 ± 110.53	0.741	0.446	0.437	0.080
PTV_*D*_98%_	1728.21 ± 201.16	1697.32 ± 219.53	1707.75 ± 210.06	1691.15 ± 156.37	1627.57 ± 272.06	0.417	0.026	0.260	0.024
PTV_CI	1.05 ± 0.07^*^	1.17 ± 0.20^*^	1.16 ± 0.16^*^	1.22 ± 0.20^*^	1.19 ± 0.18^*^	0.012	0.001	0.001	0.023

* Dimensionless value.

Additionally, the introduction of setup errors led to a significant decrease in PTV dose conformity for all plans.

Compared to the original plans, setup errors resulted in increased maximum and mean doses to all OARs (Table 3). The impact was particularly pronounced for the maximum dose to the lens, with the left lens *D*_max_ increasing by 21.3%, 32.7%, 33.8%, and 13.2% due to average setup errors and maximum errors in X, Y, and Z directions, respectively. The right lens *D*_max_ also increased by 21.3%, 31.4%, 24.5%, and 10.7%, although most of these increases were not statistically significant. The maximum doses of the left and right optic nerves, which were partially within the PTV, showed a slight but statistically significant increase. Furthermore, the number of monitor units of plans incorporating setup errors increased by 2.6%, 2.1%, 3.1%, and 2.9%, respectively.

**TABLE 3:** Statistical analysis of organs at risk (OARs) dose parameters affected by setup errors (cGy) ($\bar{x}\pm s$, cGy).

Evaluation parameter	Original plan	XYZ_mean error	X_max error	Y_max error	Z_max error	*p*‐value (XYZ_mean)	*p*‐value (X_max)	Value (Y_max)	Value (Z_max)
Left lens_*D*_max_	604.84 ± 102.01	733.94 ± 420.14	802.42 ± 245.70	809.48 ± 385.21	684.61 ± 214.95	0.195	0.010	0.027	0.080
Left lens_*D*_mean_	401.14 ± 139.84	406.49 ± 159.03	401.72 ± 78.63	400.41 ± 95.35	387.18 ± 77.85	0.907	0.985	0.983	0.692
Right lens_*D*_max_	631.23 ± 107.50	765.96 ± 398.55	829.40 ± 395.26	785.79 ± 264.57	699.05 ± 211.74	0.154	0.037	0.014	0.125
Right lens_*D*_mean_	383.30 ± 70.65	421.61 ± 158.15	423.30 ± 86.76	408.73 ± 91.37	400.23 ± 79.04	0.244	0.004	0.027	0.064
Left optic nerve_*D*_max_	2188.16 ± 40.19	2252.09 ± 107.34	2244.77 ± 65.04	2257.63 ± 83.64	2253.02 ± 78.13	0.020	0.000	0.004	0.006
Right optic nerve_*D*_max_	2196.18 ± 31.83	2258.19 ± 106.91	2248.16 ± 66.72	2262.99 ± 104.26	2259.55 ± 87.37	0.021	0.001	0.011	0.006
Optic chiasm_*D*_max_	1750.07 ± 191.44	1806.58 ± 206.40	1755.56 ± 160.10	1757.42 ± 103.12	1796.05 ± 225.71	0.005	0.897	0.882	0.369

### 3.3 CBCT‐based assessment of short‐term efficacy of IMRT–eye prominence

**FIGURE 5:**
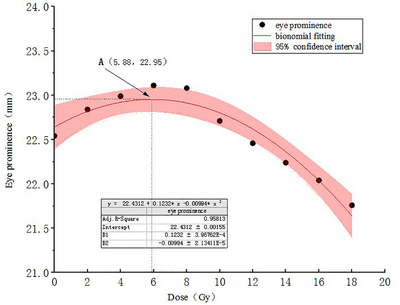
Bionomial curve fitting of thyroid‐associated ophthalmopathy (TAO) patients' eye prominence and radiotherapy dose.

The prominence of both eyes was measured on CBCT images acquired before each treatment fraction for all patients. These measurements were then fitted to a bionomial curve against the total radiation dose (Figure 5). A correlation between eye prominence and total radiotherapy dose was observed. The bionomial curve fit between these two parameters was y = 22.43 + 0.12x − (9.94 × 10^−3^) x^2^ with an *R^2^* of 0.96, indicating a good fit. The fitted curve shows that ocular prominence increased slightly with the increase of total radiotherapy dose at the initial stages, reaching a maximum value (Point A) after ≈ 5–6 fractions. Subsequently, as the number of fractions and cumulative dose increased, the degree of eye convexity gradually decreased. The mean value of eye prominence in all patients was (21.74 ± 1.74) mm before radiotherapy. Following the completion of IMRT, measured before the final fraction (i.e., after a total dose of 18 Gy), the mean prominence decreased to (20.94 ± 1.73) mm. This reduction was statistically significant (*p* < 0.05).

## 4 DISCUSSION

With the rapid development of image‐guided techniques, CBCT has played a crucial role in reducing setup errors for patients undergoing IMRT and improving the accuracy of radiation therapy. It has gradually become the benchmark for verifying radiotherapy positioning accuracy.^17^

In our study, the systematic errors were all below 1 mm, indicating high‐precision equipment performance.^18^ This also reflected the correctness of the patient fixation technique and the accuracy of the technician's operation.^7^, ^19^, ^20^ Compared with other head and neck tumors, especially nasopharyngeal carcinoma with a long target volume, the positioning accuracy in this study was higher. For example, Liu et al.^21^ used CBCT to evaluate 120 patients with nasopharyngeal carcinoma, finding setup errors of 1.5 mm, 1.3 mm, and 1.9 mm in the X, Y, and Z directions. A study by Delishaj et al.^19^ based on CBCT for patients with head and neck tumors also showed that the mean setup errors in the Z direction reached 2.0 mm.

Random errors remain the primary factor affecting setup accuracy. In our study, the random errors were consistently below 3 mm but above 1 mm, indicating that further efforts are needed to improve reproducibility. Notably, the target volume for moderate to severe TAO was relatively small and uniquely positioned near the orbital and nasal bridge areas in the anterior part of the head. This location‐specificity made it significantly different from other head and neck tumors such as brain tumors and nasopharyngeal carcinomas. Kristina et al.^22^ conducted an in‐depth study on setup errors in different regions of head and neck tumors, revealing that the nasal area exhibited the most significant setup errors. Similarly, Tangirala, using pressure sensor testing, found that the nasal bridge area showed the largest displacement.^23^ Consequently, improving setup accuracy at the nasal tip can more effectively enhance overall head positioning accuracy, including the orbital region. Additionally, patients with moderate to severe TAO may experience mild edema and other reactions in the early stages of radiotherapy, potentially affecting setup reproducibility and increasing random errors in the Z‐direction.^24^

Setup errors affect the dose distribution of radiotherapy plans. Liu et al.^25^ in their study on nasopharyngeal carcinoma, found that when setup errors exceeded 3 mm, changes in the minimum dose of plan target volume became statistically significant. In this study, under the same conditions of dose normalization in the target area, both average and maximum couch shifts due to setup errors significantly affected the *D*_max_, *D*_mean_, *D*_2%_, and *D*_50%_ of PTV. These results aligned with Kaur's^26^ findings in head and neck tumor research. This was mainly because setup errors reduced target coverage, necessitating forced dose escalation through normalization conditions, thereby increasing both *D*_max_ and *D*_mean_ in the target volume. The dose‐volume histogram also clearly showed an increase in high‐dose volumes within the target area.

Furthermore, the introduction of setup errors significantly reduced the conformity of dose distribution in all plans. Compared to the original plans, setup errors increased both *D*_max_ and *D*_mean_ for all OARs (Table 3), particularly the lens' *D*_max_. According to recent guidelines for quantitative analysis of normal tissue effects^27^ and dose constraints for OARs^28^ the *D*_max_ of the lens, as a serial organ, should be kept below 7 Gy. As the dose increases further, the probability of severe complications such as cataracts rises correspondingly, with a 50% incidence rate within 5 years at a dose of 12 Gy. Therefore, using CBCT to correct setup errors not only improved setup accuracy but also enhanced dose precision and reduced radiation exposure to normal tissues. Conversely, if the existence of setup errors was ignored during IMRT without IGRT online imaging and correction, even a random error of > 2 mm might lead to missed irradiation of the target area and high‐dose irradiation of OARs. This could subsequently compromise tumor local control rates and increase toxicity to OARs.^29^ Therefore, for patients with moderate to severe TAO, the use of CBCT to detect and correct setup uncertainties before treatment was crucial for the precise implementation of IMRT.

Regarding eye prominence, it was to some extent actually attributed to pseudo‐progression induced by low‐dose irradiation.^30^ In this study, the eye prominence did not decrease continuously but increased and then decreased with increasing dose. Durkin et al.^31^ summarized the complications of ophthalmic and adnexal induced by radiotherapy, noting that acute radiation reactions such as periorbital skin erythema could occur early in the treatment course and, as reported by Hamilton et al.^32^ were almost universal. The dose‐response study by Roth et al.^33^ also confirmed that skin erythema began to appear at very low doses. The onset of eyelid edema closely coincided with the erythematous response, which was likely associated with dose‐related obstruction of the meibomian glands. The initial edema directly manifested as an increase in eye prominence. Subsequently, as the cumulative radiation dose exerted inhibitory effects on immune lymphocytes, the eye prominence began to decrease. There was a correlation between CBCT‐measured ocular prominence and the total dose of radiotherapy received by the patients. The mean value of eye prominence reduced by 3.68% with a significant difference (*p* < 0.05) after 18 Gy of IMRT. This result further confirms the short‐term efficacy of radiotherapy in improving ocular prominence, although further validation with a larger case series is still required. Therefore, when patients in this study experienced pseudo‐progression or had doubts about the effectiveness of radiotherapy, we actively communicated with them and explained the findings based on this study's fitted results, which promptly eliminated the anxiety and psychological pressure of the patients thereby improving patient compliance with radiotherapy and ensured that all the patients adhered to the completion of the full course of radiotherapy.

Of course, there were still some limitations in this study. Although there was a certain correlation between the eye prominence measurements from 115 CBCT scans of patients and the radiotherapy dose, the relatively small sample size limited the precision in determining the strength of this correlation. Additionally, this study was limited to the short‐term efficacy assessment of ocular prominence, and further follow‐up and research are needed to assess the long‐term clinical efficacy of IMRT in moderate to severe TAO.

## 5 CONCLUSIONS

In summary, the use of CBCT image guidance to correct for setup errors in IMRT for moderate to severe TAO not only improves target conformity and coverage but also reduces doses to OARs, particularly the lenses, thereby enhancing the precision of treatment delivery. Furthermore, CBCT can provide the possibility of short‐term efficacy assessment of eye prominence in radiotherapy patients which may alleviate patient anxiety with preliminary observation thereby improving patient compliance with radiotherapy.

## AUTHOR CONTRIBUTIONS

Conception and design: Dan Kang and Shizhen Bin. Acquisition of data: Shuyun Ma and Shizhen Bin. Analysis of data: Hao Hu and Dan Kang. Writing the first draft of the manuscript: Dan Kang and Shizhen Bin. All authors contributed to the drafting and editing of the manuscript and approved the final version.

## CONFLICT OF INTEREST STATEMENT

The authors declare that they have no conflict of interest.

## ETHICS APPROVAL STATEMENT

The study was conducted in accordance with relevant guidelines and regulations. This study adhered to the Declaration of Helsinki principles and was approved by the Ethics Committee of the Third Xiangya Hospital of Central South University (approval number: 2023‐S139).

## Data Availability

The data that support the findings of this study are available from the corresponding author upon reasonable request.

## References

[1] Kahaly G , Roesler H , Kutzner J , et al. Radiotherapy for thyroid‐associated orbitopathy. Exp Clin Endocrinol Diabetes. 1999;107(Suppl. 5):S201–S207. doi:10.1055/s‐0029‐1212186 10614923 10.1055/s-0029-1212186

[2] Lutt JR , Lim LL , Phal PM , Rosenbaum JT . Seminars in arthritis and rheumatism. Orbital Inflammatory Disease. Elsevier; 2008:207–222. 10.1016/j.semarthrit.2007.06.003 17765951

[3] Wang Y , Patel A , Douglas RS . Thyroid eye disease: how a novel therapy may change the treatment paradigm. Ther Clin Risk Manag. 2019;15:1305–1318. doi:10.2147/TCRM.S193018 31814726 10.2147/TCRM.S193018 PMC6858302

[4] Zeng L , Xie X‐Q , Li C‐H , Shi H‐S , Wang F . Clinical study of the radiotherapy with EDGE accelerator in the treatment of the moderate and severe thyroid associated ophthalmopathy. Eur Rev Med Pharmacol Sci. 2019;23(8):3471–3477. 31081102 10.26355/eurrev_201904_17712

[5] Horner K , O'Malley L , Taylor K , Glenny A‐M . Guidelines for clinical use of CBCT: a review. Dentomaxillofac Radiol. 2015;44(1):20140225. doi:10.1259/dmfr.20140225 25270063 10.1259/dmfr.20140225 PMC4277440

[6] Nien H‐H , Wang L‐Y , Liao L‐J , et al. Advances in image‐guided radiotherapy in the treatment of oral cavity cancer. Cancers. 2022;14(19):4630. doi:10.3390/cancers14194630 36230553 10.3390/cancers14194630 PMC9561985

[7] Den RB , Doemer A , Kubicek G , et al. Daily image guidance with cone‐beam computed tomography for head‐and‐neck cancer intensity‐modulated radiotherapy: a prospective study. Int J Radiat Oncol Biol Phys. 2010;76(5):1353–1359. doi:10.1016/j.ijrobp.2009.03.059 19540071 10.1016/j.ijrobp.2009.03.059

[8] Cole A , Veiga C , Johnson U , D'Souza D , Lalli N , McClelland J . Toward adaptive radiotherapy for lung patients: feasibility study on deforming planning CT to CBCT to assess the impact of anatomical changes on dosimetry. Phys Med Biol. 2018;63(15):155014. doi:10.1088/1361‐6560/aad1bb 29978832 10.1088/1361-6560/aad1bb PMC6329444

[9] Bapst B , Lagadec M , Breguet R , Vilgrain V , Ronot M . Cone beam computed tomography (CBCT) in the field of interventional oncology of the liver. Cardiovasc Intervent Radiol. 2016;39:8–20. doi:10.1007/s00270‐015‐1180‐6 26178776 10.1007/s00270-015-1180-6

[10] Jin X , Hu W , Shang H , et al. CBCT‐based volumetric and dosimetric variation evaluation of volumetric modulated arc radiotherapy in the treatment of nasopharyngeal cancer patients. Radiat Oncol. 2013;8:279. doi:10.1186/1748‐717X‐8‐279 24289312 10.1186/1748-717X-8-279 PMC4222038

[11] Chatzipetros E , Tsiklakis K , Donta C , Damaskos S , Angelopoulos C . Morphological assessment of nasopalatine canal using cone beam computed tomography: a retrospective study of 124 consecutive patients. Diagnostics. 2023;13(10):1787. doi:10.3390/diagnostics13101787 37238271 10.3390/diagnostics13101787 PMC10217395

[12] Mourits MP , Prummel MF , Wiersinga WM , Koornneef L . Clinical activity score as a guide in the management of patients with Graves' ophthalmopathy. Clin Endocrinol (Oxf). 1997;47(1):9–14. doi:10.1046/j.1365‐2265.1997.2331047.x 9302365 10.1046/j.1365-2265.1997.2331047.x

[13] Hodgson NM , Rajaii F . Current understanding of the progression and management of thyroid associated orbitopathy: a systematic review. Ophthalmol Ther. 2020;9:21–33. doi:10.1007/s40123‐019‐00226‐9 31823232 10.1007/s40123-019-00226-9 PMC7054489

[14] Abboud M , Arabi A , Salti I , Geara F . Outcome of thyroid associated ophthalmopathy treated by radiation therapy. Radiat Oncol. 2011;6:1–6. doi:10.1186/1748‐717X‐6‐46 21569461 10.1186/1748-717X-6-46 PMC3108307

[15] Wambersie A . ICRU report 62, prescribing, recording and reporting photon beam therapy (supplement to ICRU report 50). ICRU News. ICRU; 1999.

[16] Gregoire V , Mackie T , De Neve W , et al. ICRU report 83: prescribing, recording, and reporting photon‐beam intensity‐modulated radiation therapy (IMRT). J ICRU. 2010;10(1):1–106.

[17] van Kranen S , van Beek S , Rasch C , van Herk M , Sonke J‐J . Setup uncertainties of anatomical sub‐regions in head‐and‐neck cancer patients after offline CBCT guidance. Int J Radiat Oncol Biol Phys. 2009;73(5):1566–1573. 19306753 10.1016/j.ijrobp.2008.11.035

[18] Van Dyk J , Battista JJ , Bauman GS . Accuracy and uncertainty considerations in modern radiation oncology. Modern Technol Radiat Oncol. 2013;3:361–412.

[19] Delishaj D , Ursino S , Pasqualetti F , et al. Set‐up errors in head and neck cancer treated with IMRT technique assessed by cone‐beam computed tomography: a feasible protocol. Radiat Oncol J. 2018;36(1):54. doi:10.3857/roj.2017.00493 29621873 10.3857/roj.2017.00493 PMC5903362

[20] Zumsteg Z , DeMarco J , Lee SP , et al. Image guidance during head‐and‐neck cancer radiation therapy: analysis of alignment trends with in‐room cone‐beam computed tomography scans. Int J Radiat Oncol Biol Phys. 2012;83(2):712–719. doi:10.1016/j.ijrobp.2011.08.001 22099037 10.1016/j.ijrobp.2011.08.001

[21] Liu J , Lyman KM , Ding Z , Zhou L . Assessment of the therapeutic accuracy of cone beam computed tomography‑guided nasopharyngeal carcinoma radiotherapy. Oncol Lett. 2019;18(2):1071–1080. doi:10.3892/ol.2020.11650 31423167 10.3892/ol.2019.10412 PMC6607348

[22] Giske K , Stoiber EM , Schwarz M , et al. Local setup errors in image‐guided radiotherapy for head and neck cancer patients immobilized with a custom‐made device. IntJ Radiat Oncol Biol Phys. 2011;80(2):582–589. doi:10.1016/j.ijrobp.2010.07.1980 20934279 10.1016/j.ijrobp.2010.07.1980

[23] Tangirala DK . Accurate location of tumor in head and neck cancer radiotherapy treatment with respect to machine isocentre. 2017.

[24] Ding Y , Ma P , Li W , et al. Effect of surgical mask on setup error in head and neck radiotherapy. Technol Cancer Res Treat. 2020;19:1533033820974021. doi:10.1177/1533033820974021 33327884 10.1177/1533033820974021 PMC7750894

[25] Liu G , Zhang S , Ma Y , et al. Effects of error on dose of target region and organs at risk in treating nasopharynx cancer with intensity modulated radiation therapy. Pakistan J Med Sci. 2016;32(1):95. 10.12669/pjms.321.9218 PMC4795898 27022353

[26] Kaur I , Rawat S , Ahlawat P , et al. Dosimetric impact of setup errors in head and neck cancer patients treated by image‐guided radiotherapy. J Med Phys. 2016;41(2):144–148. doi:10.4103/0971‐6203.181640 27217627 10.4103/0971-6203.181640 PMC4871004

[27] Bentzen SM , Constine LS , Deasy JO , et al. Quantitative analyses of normal tissue effects in the clinic (QUANTEC): an introduction to the scientific issues. Int J Radiat Oncol Biol Phys. 2010;76(3):S3–S9. doi:10.1016/j.ijrobp.2009.09.040 20171515 10.1016/j.ijrobp.2009.09.040 PMC3431964

[28] Lambrecht M , Eekers DB , Alapetite C , et al. Radiation dose constraints for organs at risk in neuro‐oncology: the European Particle Therapy Network consensus. Radiother Oncol. 2018;128(1):26–36. doi:10.1016/j.radonc.2018.05.001 29779919 10.1016/j.radonc.2018.05.001

[29] Kearney M , Coffey M , Leong A . A review of image guided radiation therapy in head and neck cancer from 2009–2019–best practice recommendations for RTTs in the clinic. Tech Innov Patient Support Radiat Oncol. 2020;14:43–50. doi:10.1016/j.tipsro.2020.02.002 32566769 10.1016/j.tipsro.2020.02.002 PMC7296359

[30] Li Y‐J , Luo Y , Xie X‐Q , et al. The efficacy of intensity modulated radiation therapy in treating thyroid‐associated ophthalmopathy and predictive factors for treatment response. Sci Rep. 2017;7(1):17533. doi:10.1038/s41598‐017‐17893‐y 29235518 10.1038/s41598-017-17893-y PMC5727475

[31] Durkin SR , Roos D , Higgs B , Casson RJ , Selva D . Ophthalmic and adnexal complications of radiotherapy. Acta Ophthalmol Scand. 2007;85(3):240–250. doi:10.1111/j.1600‐0420.2006.00822.x 17488452 10.1111/j.1600-0420.2006.00822.x

[32] Hamilton CS , Denham JW , O'Brien M , et al. Under prediction of human skin erythema at low doses per fraction by the linear quadratic model. Radiother Oncol. 1996;40:23–30. doi:10.1016/0167‐8140(96)01764‐1 8844884 10.1016/0167-8140(96)01764-1

[33] Roth J , Brown N , Catterall M , Beal A . Effects of fast neutrons on the eye. Br J Ophthalmol. 1976;60(4):236–244. doi:10.1136/bjo.60.4.236 1276111 10.1136/bjo.60.4.236 PMC1017484

